# Experimental and theoretical studies of nanofluid thermal conductivity enhancement: a review

**DOI:** 10.1186/1556-276X-6-229

**Published:** 2011-03-16

**Authors:** Clement Kleinstreuer, Yu Feng

**Affiliations:** 1 Department of Mechanical and Aerospace Engineering, NC State University, Raleigh, NC 27695-7910, USA

## Abstract

Nanofluids, *i.e.*, well-dispersed (metallic) nanoparticles at low- volume fractions in liquids, may enhance the mixture's thermal conductivity, *k*_nf_, over the base-fluid values. Thus, they are potentially useful for advanced cooling of micro-systems. Focusing mainly on dilute suspensions of well-dispersed spherical nanoparticles in water or ethylene glycol, recent experimental observations, associated measurement techniques, and new theories as well as useful correlations have been reviewed.

It is evident that key questions still linger concerning the best nanoparticle-and-liquid pairing and conditioning, reliable measurements of achievable *k*_nf _values, and easy-to-use, physically sound computer models which fully describe the particle dynamics and heat transfer of nanofluids. At present, experimental data and measurement methods are lacking consistency. In fact, debates on whether the anomalous enhancement is real or not endure, as well as discussions on what are repeatable correlations between *k*_nf _and temperature, nanoparticle size/shape, and aggregation state. Clearly, benchmark experiments are needed, using the same nanofluids subject to different measurement methods. Such outcomes would validate new, minimally intrusive techniques and verify the reproducibility of experimental results. Dynamic *k*_nf _models, assuming non-interacting metallic nano-spheres, postulate an enhancement above the classical Maxwell theory and thereby provide potentially additional physical insight. Clearly, it will be necessary to consider not only one possible mechanism but combine several mechanisms and compare predictive results to new benchmark experimental data sets.

## Introduction

A nanofluid is a dilute suspension of nanometer-size particles and fibers dispersed in a liquid. As a result, when compared to the base fluid, changes in physical properties of such mixtures occur, *e.g.*, viscosity, density, and thermal conductivity. Of all the physical properties of nanofluids, the thermal conductivity (*k*_nf_) is the most complex and for many applications the most important one. Interestingly, experimental findings have been controversial and theories do not fully explain the mechanisms of elevated thermal conductivity. In this paper, experimental and theoretical studies are reviewed for nanofluid thermal conductivity and convection heat transfer enhancement. Specifically, comparisons between thermal measurement techniques (*e.g.*, transient hot-wire (THW) method) and optical measurement techniques (*e.g.*, forced Rayleigh scattering (FRS) method) are discussed. Recent theoretical models for nanofluid thermal conductivity are presented and compared, including the authors' model assuming well-dispersed spherical nanoparticles subject to micro-mixing effects due to Brownian motion. Concerning theories/correlations which try to explain thermal conductivity enhancement for all nanofluids, not a single model can predict a wide range of experimental data. However, many experimental data sets may fit between the lower and upper mean-field bounds originally proposed by Maxwell where the static nanoparticle configurations may range from a dispersed phase to a pseudo-continuous phase. Dynamic *k*_nf _models, assuming non-interacting metallic nano-spheres, postulate an enhancement above the classical Maxwell theory and thereby provide potentially additional physical insight. Clearly, it will be necessary to consider not only one possible mechanism but combine several mechanisms and compare predictive results to new benchmark experimental data sets.

## Experimental studies

Nanofluids are a new class of heat transfer fluids by dispersing nanometer-size particles, *e.g.*, metal-oxide spheres or carbon nanotubes, with typical diameter scales of 1 to 100 nm in traditional heat transfer fluids. Such colloidal dispersions may be uniform or somewhat aggregated. Earlier experimental studies reported greater enhancement of thermal conductivity, *k*_nf_, than predicted by the classical model of Maxwell [1], known as the mean-field or effective medium theory. For example, Masuda [2] showed that different nanofluids (*i.e.*, Al_2_O_3_-water, SiO_2_-water, and TiO_2_-water combinations) generated a *k*_nf _increase of up to 30% at volume fractions of less than 4.3%. Such an enhancement phenomenon was also reported by Eastman and Choi [3] for CuO-water, Al_2_O_3_-water and Cu-Oil nanofluids, using the THW method. In the following decades, it was established that nanofluid thermal conductivity is a function of several parameters [4,5], *i.e.*, nanoparticle material, volume fraction, spatial distribution, size, and shape, as well as base-fluid type, temperature, and pH value. In contrast, other experimentalists [6-9], reported that no correlation was observed between *k*_nf _and nanofluid temperature *T*. Furthermore, no *k*_nf _enhancement above predictions based on Maxwell's effective medium theory for non-interacting spherical nanoparticles was obtained [5]. Clearly, this poses the question if nanofluids can provide greater heat transfer performance, as it would be most desirable for cooling of microsystems. Some scientists argued that the anomalous *k*_nf _enhancement data are caused by inaccuracies of thermal measurement methods, *i.e.*, mainly intrusive vs. non-intrusive techniques. However, some researchers [10,11], relying on both optical and thermal measurements, reported *k*_nf _enhancements well above classical model predictions. When comparing different measurement methods, error sources may result from the preparation of nanofluids, heating process, measurement process, cleanliness of apparatus, and if the nanoparticles stay uniformly dispersed in the base fluid or aggregate [12]. Thus, the controversy is still not over because of those uncertainties.

## Experimental measurement methods

The most common techniques for measuring the thermal conductivity of nanofluids are the transient hot-wire method [9,12-15], temperature oscillation method [16,17], and 3-ω method [18,19]. As an example of a non-intrusive (optical) technique, forced Rayleigh scattering is discussed as well.

## Transient hot-wire method

THW method is the most widely used static, linear source experimental technique for measuring the thermal conductivity of fluids. A hot wire is placed in the fluid, which functions as both a heat source and a thermometer [20,21]. Based on Fourier's law, when heating the wire, a higher thermal conductivity of the fluid corresponds to a lower temperature rise. Das [22] claimed that during the short measurement interval of 2 to 8 s, natural convection will not influence the accuracy of the results.

The relationship between thermal conductivity *k*_nf _and measured temperature *T *using the THW method is summarized as follows [20]. Assuming a thin, infinitely long line source dissipating heat into a fluid reservoir, the energy equation in cylindrical coordinates can be written as:(1)

with initial condition and boundary conditions(2a)

and(2b-c)

The analytic solution reads:(3)

where *γ *= 0.5772 is Euler's constant. Hence, if the temperature of the hot wire at time *t*_1 _and *t*_2 _are *T*_1 _and *T*_2_, then by neglecting higher-order terms the thermal conductivity can be approximated as:(4)

For the experimental procedure, the wire is heated via a constant electric power supply at step time *t*. A temperature increase of the wire is determined from its change in resistance which can be measured in time using a Wheatstone-bridge circuit. Then the thermal conductivity is determined from Eq. 4, knowing the heating power (or heat flux *q*) and the slope of the curve ln(*t*) versus *T*.

The advantages of THW method are low cost and easy implementation. However, the assumptions of an infinite wire-length and the ambient acting like a reservoir (see Eqs. 1 and 2c) may introduce errors. In addition, nanoparticle interactions, sedimentation and/or aggregation as well as natural convection during extended measurement times may also increase experimental uncertainties [19,23].

## Other thermal measurement methods

A number of improved hot-wire methods and experimental designs have been proposed. For example, Zhang [24] used a short-hot-wire method (see also Woodfield [25]) which can take into account boundary effects. Mintsa [26] inserted a mixer into his THW experimental devices in order to avoid nanoparticle aggregation/deposition in the suspensions. Ali et al. [27] combined a laser beam displacement method with the THW method to separate the detector and heater to avoid interference.

Alternative static experimental methods include the temperature oscillation method [16,17,28], micro-hot-strip method [29], steady-state cut-bar method [30], 3-ω method [18,31,32], radial heat-flow method [33], photo-thermal radiometry method [34], and thermal comparator method [19,35].

It is worth mentioning that most of the thermal measurement techniques are static or so called "bulk" methods (see Eq. 4). However, nanofluids could be used as coolants in forced convection, requiring convective measurement methods to obtain thermal conductivity data. Some experimental results of convective nanofluid heat transfer characteristics are listed in Table 1. For example, Lee [36] fabricated a microchannel, *D*_h _= 200 μm, to measure the nanofluid thermal conductivity with a modest enhancement when compared to the result obtained by the THW method. Also, Kolade et al. [37] considered 2% Al_2_O_3_-water and 0.2% multi-wall carbon nano-tube (MWCNT)-silicone oil nanofluids. By measuring the thermal conductivities of nanofluids in a convective environment, Kolade et al. [37] obtained 6% enhancement for Al_2_O_3_-water nanofluid and 10% enhancement for MWCNT-silicone oil nanofluid. Such enhancements are very modest compared to the experimental data obtained by THW methods.

**Table 1:** Summary of experimental studies on convective heat transfer properties of nanofluids

Reference	Nanofluids	Flow nature	Findings
Pak and Cho [91]	*d*_p _= 13 nm spherical Al_2_O_3_-water *d*_p _= 27 nm spherical TiO_2_-water	Tube/turbulent	Nu is 30% larger than conventional base fluid and larger than Dittus-Boelter prediction
Li and Xuan [92]	*d*_p _< 100 nm spherical Cu-water	Tube/turbulent	Nu is larger than Dittus-Boelter prediction when volume fraction *φ *> 0.5%
Wen and Ding [93]	d_p _= 27-56 nm spherical Al_2_O_3_-water	Tube/laminar	Nu > 4.36 for fully-developed pipe flow with constant wall heat flux
Ding [94]	d_p _> 100 nm rodlike carbon nanotube-water	Tube/laminar	Nu increase more than 300% at Re = 800
Heris [95]	d_p _= 20 nm spherical Al_2_O_3_-water	Tube/laminar	Nu measured is larger than Nu of pure water
Williams [49]	d_p _= 46 nm spherical Al_2_O_3_-water d_p _= 60 nm spherical ZrO_2_-water	Tube/turbulent	Nu of nanofluids can be predicted by traditional correlations and models. No abnormal heat transfer enhancement was observed.
Kolade [37]	d_p _= 40-50 nm spherical Al_2_O_3_-water rodlike carbon nanotube-oil	Tube/laminar	Nu is apparently larger than pure based fluid
Duangthongsuk [14]	d_p _= 21 nm spherical TiO_2_-water	Tube/turbulent	Pak and Cho (1998) correlation show better agreement to experimental data of Nu than Xuan and Li (2002) correlation
Rea [96]	d_p _= 50 nm spherical Al_2_O_3_-water d_p _= 50 nm spherical ZrO_2_-water	Tube/laminar	Nu of Al_2_O_3_-water nanofluid show up to 27% more than pure water, ZrO_2_-water displays much lower enhancement.
Jung [90]	d_p _= 170 nm spherical Al_2_O_3_-water d_p _= 170 nm spherical Al_2_O_3_-ethylene glycol	Rectangular microchannel/laminar	Nu increases with increasing the Reynolds number in laminar flow regime, appreciable enhancement of Nu is measured
Heris [97]	spherical Al_2_O_3_-water	Tube/laminar	Nu increases with increasing the Peclet number and *φ*, Brownian motion may play role in convective heat transfer enhancement

Actually, "convective" k_nf _values are not directly measured. Instead, wall temperature *T*_w _and bulk temperature *T*_b _are obtained and the heat transfer coefficient is then calculated as h = *q*_w_/(*T*_w _- *T*_b_). From the definition of the Nusselt number, *k*_nf _= *hD*/Nu where generally *D *is the hydraulic diameter. With *h *being basically measured and *D *known, either an analytic solution or an iterative numerical evaluation of Nu is required to calculate *k*_nf_. Clearly, the accuracy of the "convective measurement method" largely depends on the degree of uncertainties related to the measured wall and bulk temperatures as well as the computed Nusselt number.

## Optical measurement methods

In recent years, optical measurement methods have been proposed as non-invasive techniques for thermal conductivity measurements to improve accuracy [6-9,13,11,27,37]. Indeed, because the "hot wire" is a combination of heater and thermometer, interference is unavoidable. However, in optical techniques, detector and heater are always separated from each other, providing potentially more accurate data. Additionally, measurements are completed within several microseconds, *i.e.*, much shorter than reported THW-measurement times of 2 to 8 s, so that natural convection effects are avoided.

For example, Rusconi [6,38] proposed a thermal-lensing (TL) measurement method to obtain *k*_nf _data. The nanofluid sample was heated by a laser-diode module and the temperature difference was measured by photodiode as optical signals. After post-processing, the thermal conductivity values were generated, which did not exceed mean-field theory results. Similar to the TL method, FRS have been used to investigate the thermal conductivity of well-dispersed nanofluids [8,39]. Again, their results did not show any anomalous enhancement either for Au-or Al_2_O_3_-nanofluids. Also, based on their data, no enhancement of thermal conductivity with temperature was observed. In contrast, Buongiorno et al. [9] presented data agreement when using both the THW method and FRS method. Another optical technique for thermal conductivity measurements of nanofluids is optical beam deflection [7,40]. The nanofluid is heated by two parallel lines using a square current. The temperature change of nanofluids can be transformed to light signals captured by dual photodiodes. For Au-nanofluids, Putnam [7] reported significantly lower k_nf _enhancement than the data collected with the THW method.

However, other papers based on optical measurement techniques showed similar enhancement trends for nanofluid thermal conductivities as obtained with the thermal measurement methods. For example, Shaikh et al. [10] used the modern light flash technique (LFA 447) and measured the thermal conductivity of three types of nanofluids. They reported a maximum enhancement of 161% for the thermal conductivity of carbon nanotube (CNT)-polyalphaolefin (PAO) suspensions. Such an enhancement is well above the prediction of the classical model by Hamilton and Crosser [41]. Also, Schmidt et al. [13] compared experimental data for Al_2_O_3_-PAO and C_10_H_22_-PAO nanofluids obtained via the Transient Optical Grating method and THW method. In both cases, the thermal conductivities were greater than expected from classical models. Additionally, Bazan [11] executed measurements by three different methods, *i.e.*, laser flash (LF), transient plane source, and THW for PAO-based nanofluids. They concluded that the THW method is the most accurate one while the LF method lacks precision when measuring nanofluids with low thermal conductivities. Also, no correlation between thermal conductivity and temperature was observed. Clearly, materials and experimental methods employed differ from study to study, where some of the new measurement methods were not verified repeatedly [6,7]. Thus, it will be necessary for scientists to use different experimental techniques for the same nanofluids in order to achieve high comparable accuracy and prove reproducibility of the experimental results.

## Experimental observations

Nearly all experimental results before 2005 indicate an anomalous enhancement of nanofluid thermal conductivity, assuming well-dispersed nanoparticles. However, more recent efforts with refined transient hot-wire and optical methods spawned a controversy on whether the anomalous enhancement beyond the mean-field theory is real or not. Eapen et al. [5] suggested a solution, arguing that even for dilute nanoparticle suspensions *k*_nf _enhancement is a function of the aggregation state and hence connectivity of the particles; specifically, almost all experimental k_nf _data published fall between lower and upper bounds predicted by classical theories.

**Figure 1:**
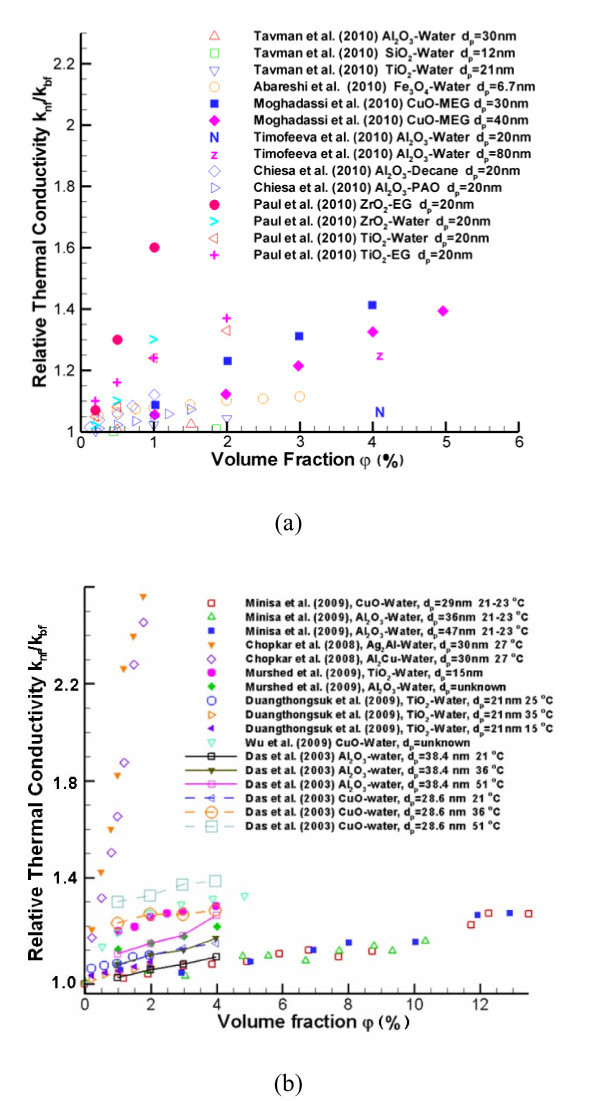
**Experimental data for the relationship between k**_**nf **_**and volume fraction**. See refs. [14,16,19,23,26,32,46-48,53,87,88].

**Figure 2:**
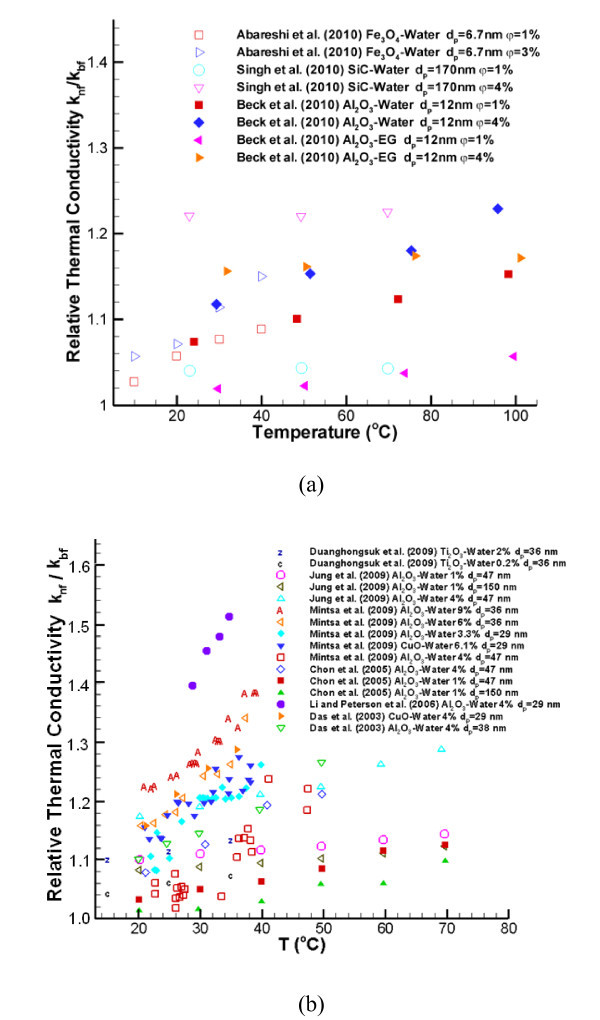
**Experimental data for the relationship between k**_**nf **_**and temperature**. See refs. [14,16,26,44,48,57,63,89,90].

In order to provide some physical insight, benchmark experimental data sets obtained in 2010 as well as before 2010 are displayed in Figures 1 and 2. Specifically, Figure 1a,b demonstrate that *k*_nf _increases with nanoparticle volume fraction. This is because of a number of interactive mechanisms, where Brownian-motion-induced micro-mixing is arguably the most important one when uniformly distributed nanoparticles can be assumed. Figure 2a,b indicate that *k*_nf _also increases with nanofluid bulk temperature. Such a relationship can be derived based on kinetics theory as outlined in Theoretical studies section. The impact of nanoparticle diameter on k_nf _is given in Figures 1 and 2 as well. Compared to older benchmark data sets [16-19], new experimental results shown in Figures 1 and 2 indicate a smaller enhancement of nanofluid thermal conductivity, perhaps because of lower experimental uncertainties. Nevertheless, discrepancies between the data sets provided by different research groups remain.

In summary, *k*_nf _is likely to improve with nanoparticle volume fraction and temperature as well as particle diameter, conductivity, and degree of aggregation, as further demonstrated in subsequent sections.

### Thermal conductivity *k*_nf _vs. volume fraction *φ*

Most experimental observations of nanofluids with just small nanoparticle volume fractions showed that *k*_nf _will significantly increase when compared to the base fluid. For example, Lee and Choi [42] investigated CuO-water/ethylene glycol nanofluids with particle diameters 18.6 and 23.6 nm as well as Al_2_O_3_-water/ethylene glycol nanofluids with particle diameters 24.4 and 38.4 nm and discovered a 20% thermal conductivity increase at a volume fraction of 4%. Wang [43] measured a 12% increase in *k*_nf _for 28-nm-diameter Al_2_O_3_-water and 23 nm CuO-water nanofluids with 3% volume fraction. Li and Peterson [44] provided thermal conductivity expressions in terms of temperature (*T*) and volume fraction (*φ*) by using curve fitting for CuO-water and Al_2_O_3_-water nanofluids. For non-metallic particles, *i.e.*, SiC-water nanofluids, Xie [45] showed a *k*_nf _enhancement effect. Recently, Mintsa [26] provided new thermal conductivity expressions for Al_2_O_3_-water and CuO-water nanofluids with particle sizes of 47, 36, and 29 nm by curve fitting their in-house experimental data obtained by the THW method. Murshed [46] measured a 27% increase in 4% TiO_2_-water nanofluids with particle size 15 nm and 20% increase for Al_2_O_3_-water nanofluids. However, Duangthongsuk [14] reported a more moderate increase of about 14% for TiO_2_-water nanofluids. Quite surprising, Moghadassi [47] observed a 50% increment of thermal conductivity for 5% CuO-monoethylene glycol (MEG) and CuO-paraffin nanofluids.

### Thermal conductivity *k*_nf _vs. temperature *T*

Das [16] systematically discussed the relationship between thermal conductivity and temperature for nanofluids, noting significant increases of *k*_nf _(*T*). More recently, Abareshi et al. [48] measured the thermal conductivity of Fe_3_O_4_-water with the THW method and asserted that *k*_nf _increases with temperature *T*. Indeed, from a theoretical (*i.e.*, kinetics) view-point, with the increment of the nanofluid's bulk temperature *T*, molecules and nanoparticles are more active and able to transfer more energy from one location to another per unit time.

In contrast, many scientists using optical measurement techniques found no anomalous effective thermal conductivity enhancement when increasing the mixture temperature [[6-9,29,30,37,49], etc.]. Additionally, Tavman et al. [32] measured SiO_2_-water, TiO_2_-water, and Al_2_O_3_-water by the 3-ω method and claimed, without showing actual data points, that there is no anomalous thermal conductivity enhancement with increment of both volume fraction and temperature. Whether anomalous enhancement relationship between *k*_nf _and temperature *T *exist or not is still open for debate.

### Dependence of *k*_nf _on other parameters

Potentially influential parameters on thermal conductivity, other than volume fraction and temperature, include pH value, type of base fluid, nanoparticle shape, degree of nanoparticle dispersion/interaction, and various additives. For example, Zhu et al. [50] showed that the pH of a nanofluid strongly affects the thermal conductivity of suspensions. Indeed, pH value influence the stability of nanoparticle suspensions and the charges of the particle surface thereby affect the nanofluid thermal conductivity. For pH equal to 8.0-9.0, the thermal conductivity of nanofluid is higher than other situations [50] Of the most common base fluids, water exhibits a higher thermal conductivity when compared to ethylene glycol (EG) for the same nanoparticle volume fraction [43,44,51-53]. However, thermal conductivity enhancement of EG-based nanofluids is stronger than for water-based nanofluids [42,43]. Different particle shapes may also influence the thermal conductivity of nanofluids. Nanoparticles with high aspect ratios seem to enhance the thermal conductivity further. For example, spherical particles show slightly less enhancement than those containing nanorods [54], while the thermal conductivity of CuO-water-based nanofluids containing shuttle-like-shaped CuO nanoparticles is larger than those for CuO nanofluids containing nearly spherical CuO nanoparticles [55]. Another parameter influencing nanofluid thermal conductivity is particle diameter. Das [16], Patel [56] and Chon [57] showed the inverse dependence of particle size on thermal conductivity enhancement, considering three sizes of alumina nanoparticles suspended in water. Beck et al. [58] and Moghadassi et al. [47] reported that the thermal conductivity will increase with the decrease of nanoparticle diameters. However, Timofeeva et al. [53] reported that *k*_nf _increases with the increment of nanoparticle diameter for SiC-water nanofluids without publishing any data. Other factors which may influence the thermal conductivity of nanofluids are sonification time [32] and/or surfactant mass fraction [32] to obtain well-dispersed nanoparticles.

For other new experimental data, Wei X. et al. [59] reported nonlinear correlation between *k*_nf _and synthesis parameters of nanoparticles as well as temperature *T*. Li and Peterson [60] showed natural convection deterioration with increase in nanoparticle volume fraction. This may be because the nanoparticle's Brownian motion smoothen the temperature gradient leading to the delay of the onset of natural convection. Also, higher viscosity of nanofluids can also induce such an effect. Wei et al. [61] claimed that the measured apparent thermal conductivity show time-dependent characteristics within 15 min when using the THW method. They suggested that measurements should be made after 15 min in order to obtain accurate data. Chiesa et al. [23] investigated the impact of the THW apparatus orientation on thermal conductivity measurements; however, that aspect was found not to be significant. Shalkevich et al. [62] reported no abnormal thermal conductivity enhancement for 0.11% and 0.00055% of gold nanoparticle suspensions, which are rather low volume fractions. Beck et al. [63] and Teng et al. [15] provided curve-fitted results based on their in-house experimental data, reflecting correlations between *k*_nf _and several parameters, *i.e.*, volume fraction, bulk temperature and particle size. Both models are easy to use for certain types of nanofluids. Ali et al. [27] proposed hot wire-laser probe beam method to measure nanofluid thermal conductivity and confirmed that particle clustering has a significant effect on thermal conductivity enhancement.

## Theoretical studies

Significant differences among published experimental data sets clearly indicate that some findings were inaccurate. Theoretical analyses, mathematical models, and associated computer simulations may provide additional physical insight which helps to explain possibly anomalous enhancement of the thermal conductivity of nanofluids.

## Classical models

The static model of Maxwell [1] has been used to determine the effective electrical or thermal conductivity of liquid-solid suspensions of monodisperse, low-volume-fraction mixtures of spherical particles. Hamilton and Crosser [41] extended Maxwell's theory to non-spherical particles. For other classical models, please refer to Jeffery [64], Davis [65] and Bruggeman [66] as summarized in Table 2. The classical models originated from continuum formulations which typically involve only the particle size/shape and volume fraction and assume diffusive heat transfer in both fluid and solid phases [67]. Although they can give good predictions for micrometer or larger-size multiphase systems, the classical models usually underestimate the enhancement of thermal conductivity increase of *nanofluids *as a function of volume fraction. Nevertheless, stressing that nanoparticle aggregation is the major cause of *k*_nf _enhancement, Eapen et al. [5] revived Maxwell's lower and upper bounds for the thermal conductivities of dilute suspensions (see also the derivation by Hashin and Shtrikman [68]). While for the lower bound, it is assumed that heat conducts through the mixture path where the nanoparticles are well dispersed, the upper bound is valid when connected/interacting nanoparticles are the dominant heat conduction pathway. The effect of particle contact in liquids was analyzed by Koo et al. [69], *i.e.*, actually for CNTs, and successfully compared to various experimental data sets. Their stochastic model considered the CNT-length as well as the number of contacts per CNT to explain the nonlinear behavior of *k*_nf _with volume fraction.

**Table 2:** Classical models for effective thermal conductivity of mixtures

Models	Expressions	Remarks
Maxwell		Spherical particles
Hamilton-Crosser		*n *= 3 for spheres *n *= 6 for cylinders
Jeffrey		Spherical particles
Davis		High-order terms represent pair interaction of randomly dispersed sphere
Lu-Lin		Spherical and non-spherical particles

## Dynamical models and comparisons with experimental data

When using the classical models, it is implied that the nanoparticles are stationary to the base fluid. In contrast, dynamic models are taking the effect of the nanoparticles' random motion into account, leading to a "micro-mixing" effect [70]. In general, anomalous thermal conductivity enhancement of nanofluids may be due to:

• Brownian-motion-induced micro-mixing;

• heat-resistance lowering liquid-molecule layering at the particle surface;

• higher heat conduction in metallic nanoparticles;

• preferred conduction pathway as a function of nanoparticle shape, *e.g.*, for carbon nanotubes;

• augmented conduction due to nanoparticle clustering.

Up front, while the impact of micro-scale mixing due to Brownian motion is still being debated, the effects of nanoparticle clustering and preferred conduction pathways also require further studies.

Oezerinc et al. [71] systematically reviewed existing heat transfer mechanisms which can be categorized into conduction, nano-scale convection and/or near-field radiation [22], thermal waves propagation [67,72], quantum mechanics [73], and local thermal non-equilibrium [74].

For a better understanding of the micro-mixing effect due to Brownian motion, the works by Leal [75] and Gupte [76] are of interest. Starting with the paper by Koo and Kleinstreuer [70], several models stressing the Brownian motion effect have been published [22]. Nevertheless, that effect leading to micro-mixing was dismissed by several authors. For example, Wang [43] compared Brownian particle diffusion time scale and heat transfer time scale and declared that the effective thermal conductivity enhancement due to Brownian motion (including particle rotation) is unimportant. Keblinski [77] concluded that the heat transferred by nanoparticle diffusion contributes little to thermal conductivity enhancement. However, Wang [43] and Keblinski [77] failed to consider the surrounding fluid motion induced by the Brownian particles.

**Figure 3:**
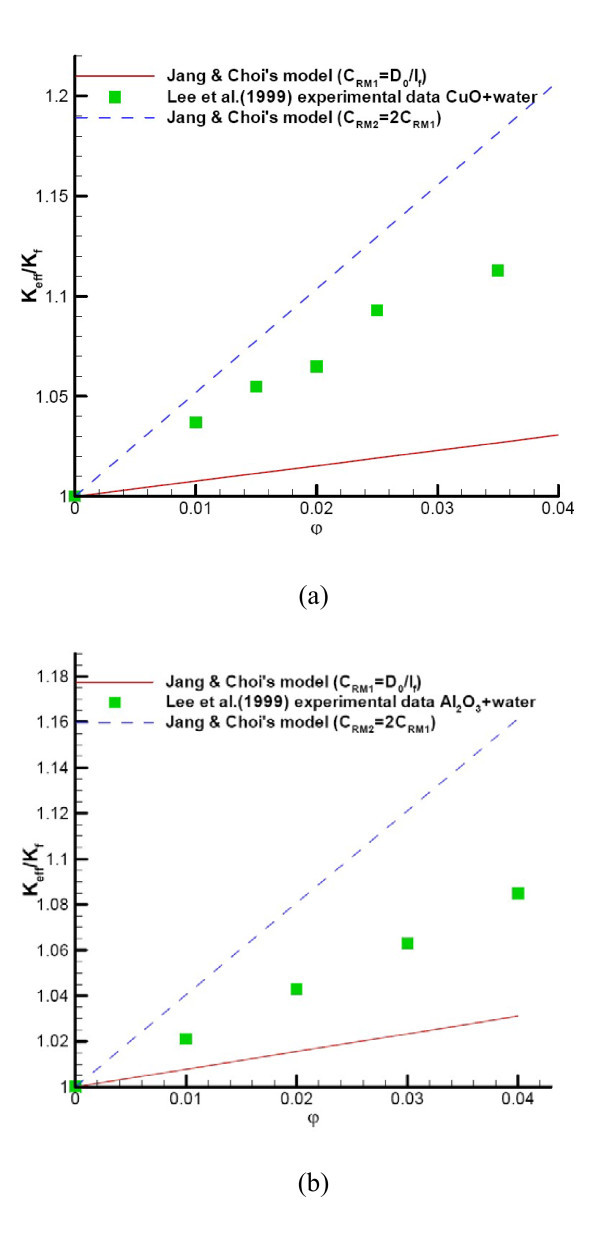
**Comparison of experimental data**. (**a**) Comparison of the experimental data for CuO-water nanofluids with Jang and Choi's model [78] for different random motion velocity definitions [80]. (**b**) Comparison of the experimental data for Al_2_O_3_-water nanofluids with Jang and Choi's model [78] for different random motion velocity definitions [80].

Incorporating indirectly the Brownian-motion effect, Jang and Choi [78] proposed four modes of energy transport where random nanoparticle motion produces a convection-like effect at the nano-scale. Their effective thermal conductivity is written as:(5)

where *C*_1 _is an empirical constant and *d*_bf _is the base fluid molecule diameter. Re_dp _is the Reynolds number, defined as:(6)

with(7)

where *D *is the nanoparticle diffusion coefficient, *κ*_Boltzmann _= 1.3807e-23 J/K is the Boltzmann constant,  is the root mean square velocity of particles and *λ*_bf _is the base fluid molecular mean free path. The definition of  (see Eq. 7b) is different from Jang and Choi's 2006 model [79]. The arbitrary definitions of the coefficient "random motion velocity" brought questions about the model's generality [78]. Considering the model by Jang and Choi [78], Kleinstreuer and Li [80] examined thermal conductivities of nanofluids subject to different definitions of "random motion velocity". The results heavily deviated from benchmark experimental data (see Figure 3a,b), because there is no accepted way for calculating the random motion velocity. Clearly, such a rather arbitrary parameter is not physically sound, leading to questions about the model's generality [80].

Prasher [81] incorporated semi-empirically the random particle motion effect in a multi-sphere Brownian (MSB) model which reads:(8)

**Figure 4:**
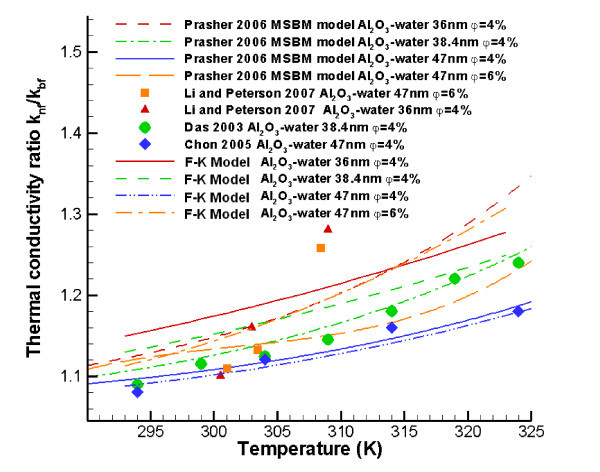
**Comparisons between Prasher's model **[81]**, the F-K model **[86]**, and benchmark experimental data **[16,44,57].

Here, Re is defined by Eq. 7a, *α *= 2*R*_b_*k*_*m*_/*d*_p _is the nanoparticle Biot number, and *R*_b _= 0.77 × 10^-8 ^Km^2^/W for water-based nanofluids which is the so-called thermal interface resistance, while *A *and *m *are empirical constants. As mentioned by Li [82] and Kleinstreuer and Li [80], the MSB model fails to predict the thermal conductivity enhancement trend when the particle are too small or too large. Also, because of the need for curve-fitting parameters *A *and *m*, Prasher's model lacks generality (Figure 4).

Kumar [83] proposed a "moving nanoparticle" model, where the effective thermal conductivity relates to the average particle velocity which is determined by the mixture temperature. However, the solid-fluid interaction effect was not taken into account.

Koo and Kleinstreuer [70] considered the effective thermal conductivity to be composed of two parts:(9)

where *k*_static _is the static thermal conductivity after Maxwell [1], *i.e.*,(10)

Now, *k*_Brownian _is the enhanced thermal conductivity part generated by midro-scale convective heat transfer of a particle's Brownian motion and affected ambient fluid motion, obtained as Stokes flow around a sphere. By introducing two empirical functions *β *and *f*, Koo [84] combined the interaction between nanoparticles as well as temperature effect into the model and produced:(11)

Li [82] revisited the model of Koo and Kleinstreuer (2004) and replaced the functions *β *and *f*(*T*,*φ*) with a new g-function which captures the influences of particle diameter, temperature and volume fraction. The empirical g-function depends on the type of nanofluid [82]. Also, by introducing a thermal interfacial resistance *R*_f _= 4e - 8 km^2^/W the original *k*_p _in Eq. 10 was replaced by a new *k*_p,eff _in the form:(12)

Finally, the KKL (Koo-Kleinstreuer-Li) correlation is written as:(13)

where *g*(*T*,*φ*,*d*_p_) is:(14)

**Figure 5:**
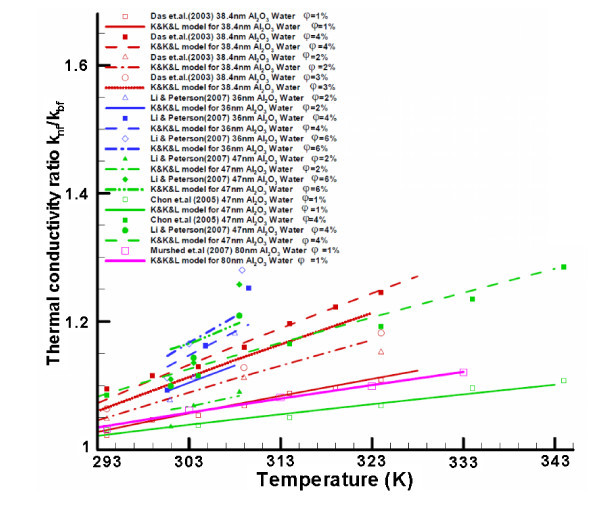
**Comparisons between KKL model and benchmark experimental data **[82].

The coefficients *a*-*k *are based on the type of particle-liquid pairing [82]. The comparison between KKL model and benchmark experimental data are shown in Figure 5.

**Figure 6:**
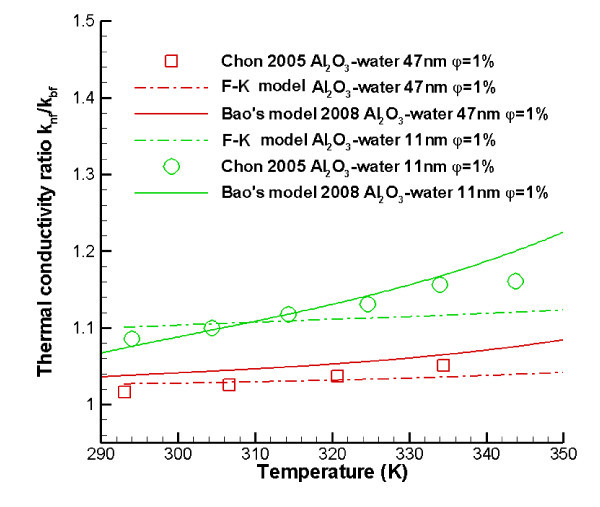
**Comparisons between Bao's model, F-K model and benchmark experimental data**.

In a more recent paper dealing with the Brownian motion effect, Bao [85] also considered the effective thermal conductivity to consist of a static part and a Brownian motion part. In a deviation from the KKL model, he assumed the velocity of the nanoparticles to be constant, and hence treated the ambient fluid around nanoparticle as steady flow. Considering convective heat transfer through the boundary of the ambient fluid, which follows the same concept as in the KKL model, Bao [85] provided an expression for Brownian motion thermal conductivity as a function of volume fraction *φ*, particle Brownian motion velocity *v*_p _and Brownian motion time interval *τ*. Bao asserted that the fluctuating particle velocity *v*_p _can be measured and *τ *can be expressed via a velocity correlation function based on the stochastic process describing Brownian motion. Unfortunately, he did not consider nanoparticle interaction, and the physical interpretation of *R*(*t*) is not clear. The comparisons between Bao's model and experimental data are shown in Figure 6. For certain sets of experimental data, Bao's model shows good agreement; however, it is necessary to select a proper value of a matching constant *M *which is not discussed in Bao [85].

Feng and Kleinstreuer [86] proposed a new thermal conductivity model (labeled the F-K model for convenience). Enlightened by the turbulence concept, *i.e.*, just random quantity fluctuations which can cause additional fluid mixing and not turbulence structures such as diverse eddies, an analogy was made between random Brownian-motion-generated fluid-cell fluctuations and turbulence. The extended Langevin equation was employed to take into account the inter-particle potentials, Stokes force, and random force.(15)

Combining the continuity equation, momentum equations and energy equation with Reynolds decompositions of parameters, *i.e.*, velocity and temperature, the F-K model can be expressed as:(16)

The static part is given by Maxwell's model [1], while the micro-mixing part is given by:(17)

**Figure 7:**
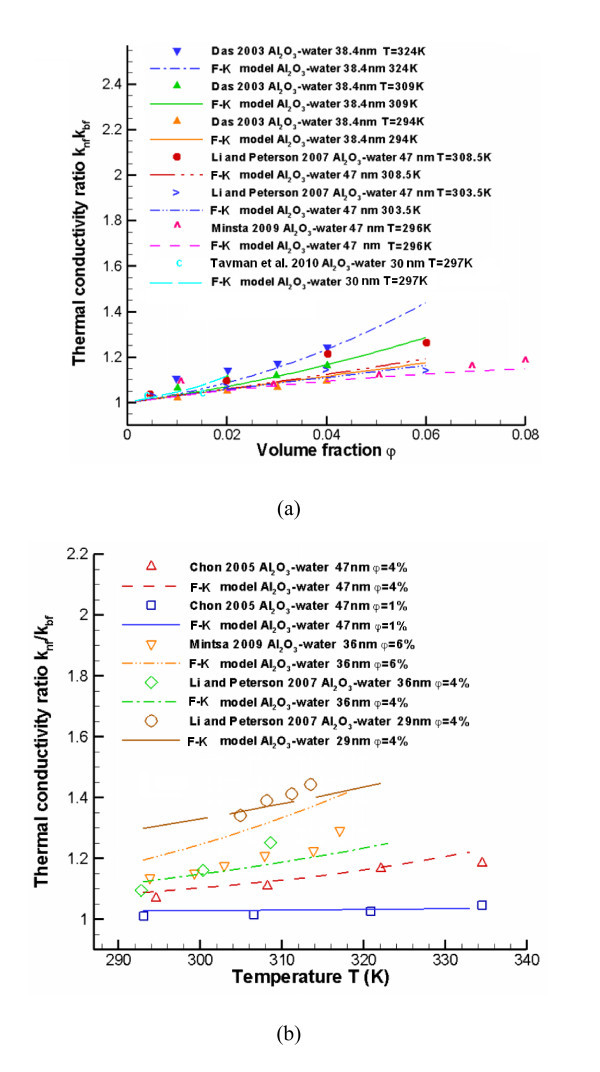
**Comparisons between the F-K model and benchmark experimental data**.

The comparisons between the F-K model and benchmark experimental data are shown in Figures 4, 6, 7a,b. Figure 7a also provides comparisons between F-K model predictions and two sets of newer experimental data [26,32]. The F-K model indicates higher *k*_nf _trends when compared to data by Tavman and Turgut [32], but it shows a good agreement with measurements by Mintsa et al. [26]. The reason may be that the volume fraction of the nanofluid used by Tavman and Turgut [32] was too small, *i.e.*, less than 1.5%. Overall, the F-K model is suitable for several types of metal-oxide nanoparticles (20 <*d*_*p *_< 50 nm) in water with volume fractions up to 5%, and mixture temperatures below 350 K.

## Summary and future work

Nanofluids, *i.e.*, well-dispersed metallic nanoparticles at low volume fractions in liquids, enhance the mixture's thermal conductivity over the base-fluid values. Thus, they are potentially useful for advanced cooling of micro-systems. Still, key questions linger concerning the best nanoparticle-and-liquid pairing and conditioning, reliable measurements of achievable *k*_nf _values, and easy-to-use, physically sound computer models which fully describe the particle dynamics and heat transfer of nanofluids. At present, experimental data and measurement methods are lacking consistency. In fact, debates are still going on whether the anomalous enhancement is real or not, and what are repeatable correlations between *k*_nf _and temperature, nanoparticle size/shape, and aggregation state. Clearly, additional benchmark experiments are needed, using the same nanofluids subject to different measurement methods as well as variations in nanofluid characteristics. This would validate new, minimally intrusive techniques and verify the reproducibility of experimental results.

Concerning theories/correlations which try to explain thermal conductivity enhancement for all nanofluids, not a single model can predict a wide range of experimental observations. However, many experimental data sets may fit between the lower and upper mean-field bounds originally proposed by Maxwell [1], where the static nanoparticle configurations may range between the two extremes of a dispersed phase to a continuous phase. Dynamic *k*_nf _models postulate an enhancement above the classic Maxwell theory and thereby provide additional physical insight. Clearly, it will be necessary to consider not only one possible mechanism but combine several mechanisms and compare predictive results to new benchmark experimental data sets.

## Competing interests

The authors declare that they have no competing interests.

## Authors' contributions

YF conducted the extensive literature review and CK wrote the article. Both authors read and approved the final manuscript.
