# Experimental and theoretical studies of nanofluid thermal conductivity enhancement: a review

**DOI:** 10.1186/1556-276X-6-439

**Published:** 2011-07-01

**Authors:** Clement Kleinstreuer, Yu Feng

**Affiliations:** 1 Department of Mechanical and Aerospace Engineering, NC State University, Raleigh, NC 27695-7910, USA

## Abstract

Correction to Kleinstreuer C, Feng Y: **Experimental and theoretical studies of nanofluid thermal conductivity enhancement: a review**. *Nanoscale Research Letters *2011, **6**:229.

## Correction

The authors would like to correct the following statement within section 'Dependence of *k*_nf _on other parameters' in our published paper [1]:

However, based on experimental data, Timofeeva et al. [53] reported that k_nf _increases with the incensement of nanoparticle diameter for SiC-water nanofluid which can be explained by the change of Kapitza resistance contribution for particles with different diameters and interface areas.
